# Crosstalk: keratinocytes and immune cells in psoriasis

**DOI:** 10.3389/fimmu.2023.1286344

**Published:** 2023-11-09

**Authors:** Masahiro Kamata, Yayoi Tada

**Affiliations:** Department of Dermatology, Teikyo University School of Medicine, Tokyo, Japan

**Keywords:** psoriasis, crosstalk, immune cell, skin cell, keratinocyte

## Abstract

In the past, psoriasis was considered a skin disease caused only by keratinocyte disorders. However, the efficacy of immunosuppressive drugs and biologics used to treat psoriasis proves that psoriasis is an immune-mediated disease. Indeed, a variety of immune cells are involved in the pathogenesis of psoriasis, including dendritic cells, Th17 cells, and resident memory T cells. Furthermore, keratinocytes play a role in the development of psoriasis as immune cells by secreting antibacterial peptides, chemokines, tumor necrosis factor-α, interleukin (IL)-36, and IL-23. These immune cells and skin cells interact and drive the aberrant differentiation and proliferation of keratinocytes. This crosstalk between keratinocytes and immune cells critical in the pathogenesis of psoriasis forms an inflammatory loop, resulting in the persistence or exacerbation of psoriasis plaques.

## 1 Introduction

Psoriasis is a chronic inflammatory skin disease clinically characterized by indurated scaly erythema and pathologically by abnormal differentiation and proliferation of keratinocytes. Therefore, in the past, psoriasis was considered a skin disease caused only by keratinocyte disorders. However, reports of psoriasis successfully treated with cyclosporine have altered our understanding of the pathogenesis of psoriasis. In addition, the efficacy of immunosuppressive drugs and biologics used to treat psoriasis proves that psoriasis is an immune-mediated disease (1, 2).

To date, many studies have revealed how a variety of immune cells are involved in the pathogenesis of psoriasis. Furthermore, keratinocytes are not only the consequences of immune reactions (namely, phenotype), but also themselves play a role in the development of psoriasis as immune cells. These immune cells and keratinocytes interact, consequently driving the aberrant differentiation and proliferation of keratinocytes.

In this review article, we focus on this crosstalk mechanism and discuss its importance in the pathogenesis of psoriasis.

## 2 Crosstalk: immune cells to keratinocytes

**Figure 1:**
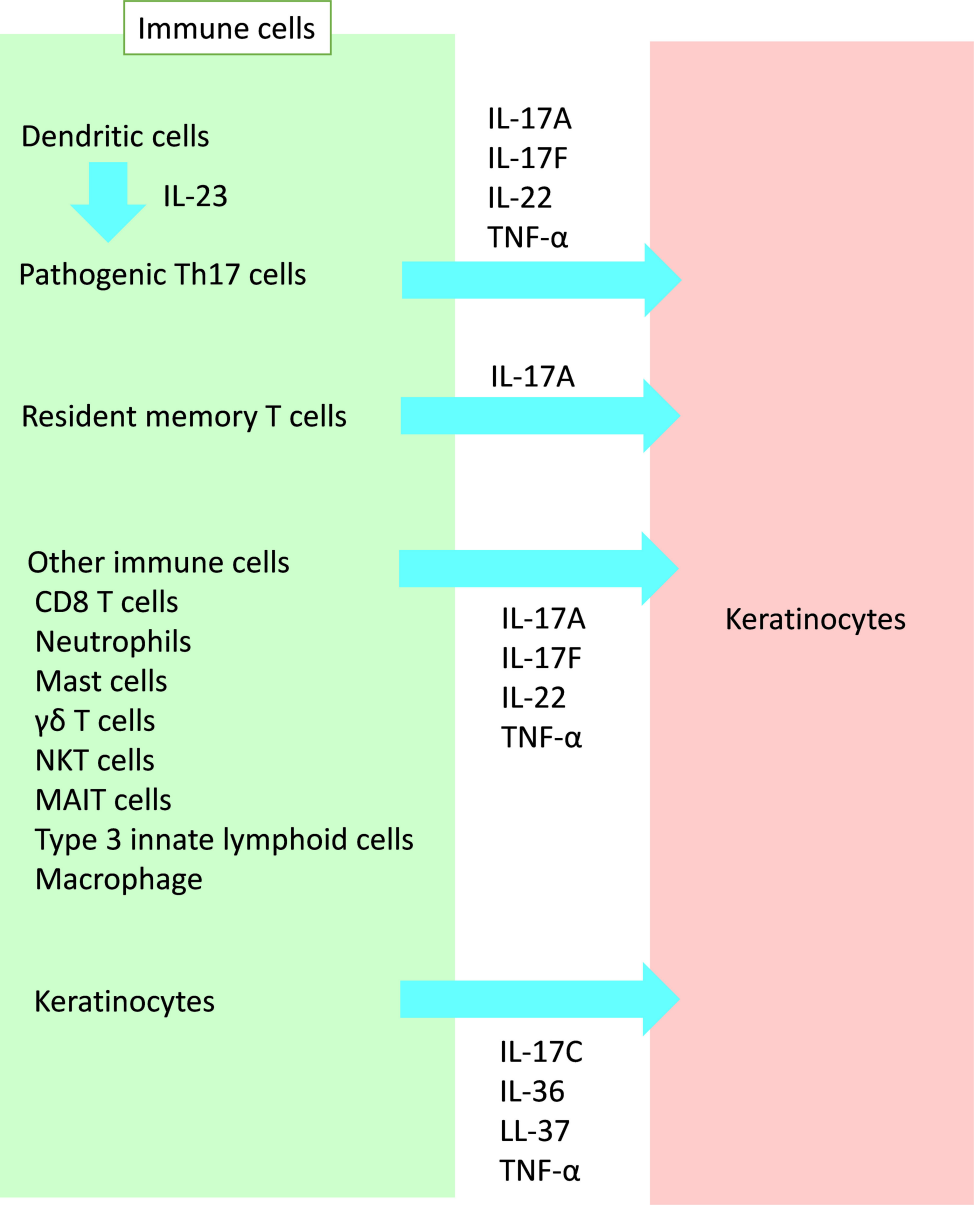
Crosstalk from immune cells to keratinocytes in psoriasis. A variety of immune cells affect keratinocytes in psoriasis. IL, interleukin; TNF, tumor necrosis factor; NKT, natural killer T; MAIT, mucosal-associated invariant T.

In the pathogenesis of psoriasis, interleukin (IL)-17 plays a key role. Moreover, IL-17 induces the proliferation and abnormal differentiation of keratinocytes (3). Keratinocytes simulated with IL-17 and tumor necrosis factor (TNF)-α produce various inflammatory cytokines, chemokines, and antibacterial peptides (AMPs) (4–6), as discussed later. IL-22 also activates keratinocytes, resulting in the proliferation and production of these inflammatory substances (7–10). In this section, we focus on immune cells that affect keratinocytes in psoriasis (**Figure 1**).

### 2.1 Pathogenic Th17 cells induced by dendritic cells

Th17 cells play a pivotal role in the pathogenesis of psoriasis. Murine studies have revealed that transforming growth factor (TFG)-β and IL-6 are required to activate a unique transcription factor known as retinoid-related orphan receptor-γt (RORγt). RORγt in association with other transcription factors, increases both IL-23R and IL-17A in Th17 cells. Subsequent exposure of IL-23 to developing Th17 cells enhances Th17 cytokines, including IL-17 (11). Human Th17 cells produce mainly IL-17A, IL-17F, and IL-22 in addition to TNF-α (12, 13). Their cytokines drive keratinocytes to their aberrant differentiation and proliferation, as well as producing pro-inflammatory substances. IL-23 promotes Th17 cells to become highly pathogenic. It also regulates the development and maintenance of the Th17 population (14–16). The main source of IL-23 is thought to be inflammatory dendritic cells (DC), as described in our previous review article (16), including TNF-α and inducible nitric oxide synthase (iNOS)-producing DC (Tip-DC) and slanDC.

### 2.2 Resident memory T cells

Recently, skin resident memory T (Trm) cells have recieved attention, especially as the cells contributing to relapse or Köbner phenomenon (3, 17–20). In resolved psoriatic skin lesions, a population of Trm cells are observed, which are responsible for local relapse of psoriasis (17–19, 21–23). Epidermal CD8^+^CD103^+^ Trm cells are considered to be one of the major immune cells in resolved skin and are capable of IL-17A (22, 24–26). Gallais Sérézal et al. confirmed through NanoString analysis that CD49a^−^CD103^+^CD8^+^ Trm cells were capable of triggering psoriasiform tissue response (27). These results suggest that IL-17−producing CD49a^−^CD103^+^CD8^+^ Trm cells are responsible for psoriasis relapse (20, 28, 29).

In addition to skin Trm cells, memory-like γδT cells (30), and skin structural cells with inflammatory memory (31, 32) could be involved in psoriasis relapse (20).

### 2.3 Other immune cells producing IL-17A

In addition to Th17 cells, IL-17A is produced by various cells of the innate and adaptive immune systems (11). CD8^+^ IL-17-producing T cells are observed in psoriatic lesions, and they produce both Th1- and Th17-related cytokines, including interferon (IFN)-γ, TNF-α, IL-17A, IL-21, and IL-22 (33–35).

Since neutrophils and mast cells staining positive for IL-17 were identified at higher densities than IL-17^+^ T cells in psoriatic lesions, neutrophils and mast cells are considered other significant potential sources of IL-17A in psoriasis (36–38). However, whether these cells synthesize and secrete IL-17A or whether positive staining represents cytokine uptake has yet to be determined (11). Mashiko et al. reported that human mast cells are major IL-22 producers in patients with psoriasis (39). Further investigation is needed to elucidate the role of mast cells and neutrophils in the pathogenesis of psoriasis.

IL-17A and IL-17F are also secreted by innate immune cells, such as group 3 innate lymphoid cells (ILC3s), and innate-like lymphocytes (ILLs), such as γδT cells, mucosal-associated invariant T (MAIT) cells, and natural killer T (NKT) cells (40–45).

Under the condition of abundant IL-23 in psoriasis lesional skin, some macrophages may produce IL-17A, IL-22 and IFN-γ in addition to TNF-α as described in our previous review article (16).

### 2.4 Keratinocytes

Keratinocytes also act as immune cells. Some cytokines secreted by keratinocytes, including IL-17C and IL-36, act on keratinocytes in an autocrine way (46). IL-36 cytokines, such as IL-36α/β/γ, are produced by keratinocytes following stimulation by TNF-α, IL-17A, IL-22, and IL-1β. IL-36 stimulates keratinocytes to produce TNF-α and IL-17C (47, 48). IL-17C is expressed by (and acts on) epithelial cells (49). Keratinocytes, the main producers of (and responders to) IL-17C in the skin, contribute to psoriatic inflammation (50–52). IL-17C has been identified as a functional regulator of the initial psoriatic cytokine network, suggesting its role during the early stages of psoriatic inflammation, or the “priming” for plaque formation (53).

Cathelicidins are a class of AMPs. LL-37, one of cathelicidins, produced by skin injury and bacterial infection, activates toll-like receptor (TLR)8 in keratinocytes and induces IL-17C through the induction of IL-36γ (47). Inhibition of IL-17 results in normalization of IL-36γ and IL-17C to levels associated with non-lesional skin (54).

## 3 Crosstalk: keratinocytes to immune cells

**Figure 2:**
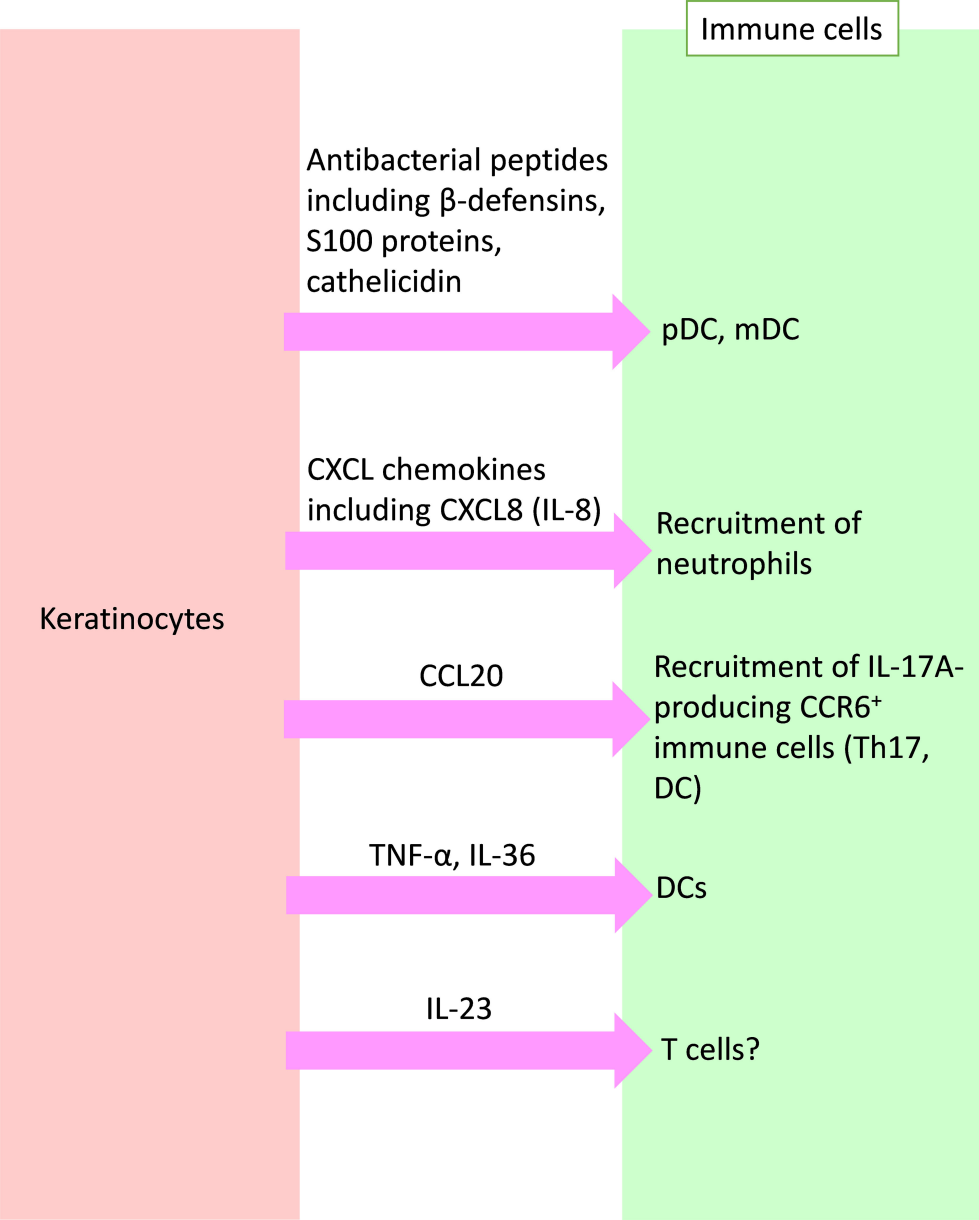
Crosstalk from keratinocytes to immune cells in psoriasis. Activated keratinocytes produce antibacterial peptides, chemokines, and inflammatory cytokines, and affect immune cells in psoriasis. pDC, plasmacytoid dendritic cell; mDC, myeloid DC; CXCL, C-X-C motif chemokine ligand; CCL, C-C motif chemokine ligand; IL, interleukin; TNF, tumor necrosis factor.

Reciprocally, keratinocytes also produce various substances that affect immune cells. In this section, we focus on these substances and their effects on immune cells (**Figure 2**).

### 3.1 Antibacterial peptides, including β-defensins, S100 proteins and cathelicidin

In non-lesional skin in psoriasis patients, trauma, injury, infection, or medication causes the production of various autoantigens from stressed or damaged keratinocytes (20, 55, 56). Among them, cationic AMPs [including LL-37, human beta-defensin (hBD)-2, and hBD-3], develop with DNA or RNA to form multimeric AMP−nucleic acid complexes, which induce the production of interferon (IFN)-α and IFN-β through TLR7 or 8 in plasmacytoid dendritic cells (pDCs) or increase the amounts of IL-6 and TNF-α by myeloid dendritic cells (mDCs) (20, 57, 58). IL-6, together with TGF-β, drives naïve T cell differentiation into Th17 cells, as described above. IFN-α and TNF-α further activate mDCs to produce IL-12 and IL-23 (16, 20, 59, 60). This process could be involved in the mechanism underlying Köbner phenomenon in psoriasis (3, 61).

In psoriatic lesions, various AMPs such as hBD-2, hBD-3, S100 proteins, and cathelicidin, are also highly expressed (15, 62, 63). hBD-2 and hBD-3 are induced by TNF-α and IFN-γ in keratinocytes (64, 65). hBD-2 is also induced by IL-17A and IL-22 (66). S100 proteins, such as S100A7 (psoriasin), S100A8 (calgranulin A), S100A9 (calgranulin B), S100A12 (calgranulin C), and S100A15, are abundantly expressed in psoriatic lesions, and some are elevated in the serum of psoriatic patients (67).

In a study by Liang et al., IL-22 in conjunction with IL-17A or IL-17F synergistically induced the expression of hBD-2 and S100A9 and additively enhanced the expression of S100A7 and S100A8 in keratinocytes (68). S100A7 may also havechemotactic potential in psoriasis (15, 69). The LL-37 high expression in the psoriatic epidermis may also accelerate inflammation through its capacity to enable pDC to recognize self-DNA via TLR9 (58). These AMPs affect various immune cells resulting in triggering, sustaining, and/or exacerbating psoriatic inflammation.

### 3.2 Chemokines, including CXCL1, CXCL2, CXCL8 (IL-8), and CCL20

Keratinocytes stimulated with IL-17 showed increased expression of multiple chemokines, including C-X-C motif chemokine ligand (CXCL)1, CXCL2, CXCL3, CXCL5, CXCL8 (IL-8), and C-C motif chemokine ligand (CCL)20 (4, 70–72). The CXCL chemokines likely attract neutrophils to the psoriatic epidermis (3, 11). CCL20 may recruit CCR6^+^ cells, including Th17 and dendritic cells, to the skin (70). Inhibition of IL-17 normalizes expression of CXCL1, CXCL8 (IL-8), and CCL20 to the levels associated with non-lesional skin (54).

### 3.3 TNF-α, and IL-36

Keratinocytes stimulated with TNF-α, IL-17A, IL-22, and IL-1β produce IL-36 (46). IL-36 stimulates keratinocytes themselves to produce TNF-α and IL-17C (47, 48). TNF-α activates mDCs, leading to production of IL-23. IL-36 drives IFN-α production in pDCs, as well as IL-1β, IL-6, and IL-23 production in mDCs (46). These cytokines secreted by keratinocytes form an aggravating inflammatory loop in the pathogenesis of psoriasis.

### 3.4 IL-23

Several reports indicate that keratinocytes produce IL-23. Moreover, immunostaining of psoriatic lesions revealed enhanced expression of IL-23 in keratinocytes (73, 74). Park et al., using publicly available single-cell RNA sequencing data from human samples, revealed that IL-23 expression was detectable in psoriatic keratinocytes as well as DCs (75). Kelemen et al. reported that psoriasis-associated inflammatory conditions induced IL-23 mRNA expression in normal huma epidermal keratinocytes (76). Li et al., using a genetic mouse model, showed that keratinocyte-produced IL-23 was sufficient to cause chronic skin inflammation with an IL-17 profile and that epigenetic control of IL-23 expression in keratinocytes was important for chronic skin inflammation (77). However, whether the expression of IL-23 in keratinocytes in psoriasis contributes to the development of psoriasis remains to be elucidated.

## 4 Effect of IL-17 or IL-23 inhibition on immune cells and keratinocytes in psoriasis

As mentioned above, IL-23 and IL-17 play important roles in the pathogenesis of psoriasis. Indeed, biologics, including IL-23 inhibitors and IL-17 inhibitors, greatly impact keratinocytes and immune cells in psoriasis.

Secukinumab (an anti-IL-17 antibody) and guselkumab (an anti-IL-23 antibody) decrease the frequencies of inflammatory monocyte-like cells, inflammatory DC-like cells, and CD4^+^CD49a^-^CD103^-^ T cells (78). Furthermore, bimekizumab (an anti-IL17A/F antibody) induces normalization of keratinocyte-related gene products, including CXCL1, CXCL8, CCL20, IL-36γ, and IL-17C, to levels associated with non-lesional skin (54). Krueger et al. reported that secukinumab caused reductions in critical downstream targets of IL-17A in the skin, including the AMPs (DEFB4A/β-defensin 2 and the S100 family) in addition to reductions in IL-23 and IL-17 in transcriptomic analyses (79). Inhibition of IL-23 or TNF-α also caused reductions in the gene expression of Th17-induced mediators by keratinocytes, including antimicrobial peptides (80, 81). Mehta et al. reported that inhibition of IL-23 reduced memory T cells while maintaining regulatory T cells, and vice versa for secukinumab (78). Furthermore, Whiley also revealed that clinical anti-IL-23 therapy depleted IL-17-producing Trm cells from the skin of patients with psoriasis (82).

## 5 Conclusion

In psoriasis, a variety of immune cells activate keratinocytes (mainly through Th17 cytokines), resulting in their abnormal differentiation and proliferation. Activated keratinocytes produce AMPs, chemokines, and various cytokines, which cause further inflammation and the recruitment of inflammatory cells. In addition, keratinocytes activate themselves by producing IL-36, IL-17C, and TNF-α. The crosstalk between immune cells and keratinocytes contributes to the development and maintenance of psoriasis.

## Author contributions

MK: Conceptualization, Writing – original draft. YT: Supervision, Validation, Writing – review & editing, Conceptualization.
